# Physiology of Vitamin D—Focusing on Disease Prevention

**DOI:** 10.3390/nu16111666

**Published:** 2024-05-29

**Authors:** Sunil J. Wimalawansa

**Affiliations:** Cardiometabolic & Endocrine Institute, North Brunswick, NJ 08902, USA; suniljw@hotmail.com

**Keywords:** aging, 25(OH)D, calcifediol, calcitriol, cardiovascular diseases, hypovitaminosis D, epidemiology, human diseases, morbidity and mortality, prevention and treatment, public health

## Abstract

Vitamin D is a crucial micronutrient, critical to human health, and influences many physiological processes. Oral and skin-derived vitamin D is hydroxylated to form calcifediol (25(OH)D) in the liver, then to 1,25(OH)_2_D (calcitriol) in the kidney. Alongside the parathyroid hormone, calcitriol regulates neuro-musculoskeletal activities by tightly controlling blood-ionized calcium concentrations through intestinal calcium absorption, renal tubular reabsorption, and skeletal mineralization. Beyond its classical roles, evidence underscores the impact of vitamin D on the prevention and reduction of the severity of diverse conditions such as cardiovascular and metabolic diseases, autoimmune disorders, infection, and cancer. Peripheral target cells, like immune cells, obtain vitamin D and 25(OH)D through concentration-dependent diffusion from the circulation. Calcitriol is synthesized intracellularly in these cells from these precursors, which is crucial for their protective physiological actions. Its deficiency exacerbates inflammation, oxidative stress, and increased susceptibility to metabolic disorders and infections; deficiency also causes premature deaths. Thus, maintaining optimal serum levels above 40 ng/mL is vital for health and disease prevention. However, achieving it requires several times more than the government’s recommended vitamin D doses. Despite extensive published research, recommended daily intake and therapeutic serum 25(OH)D concentrations have lagged and are outdated, preventing people from benefiting. Evidence suggests that maintaining the 25(OH)D concentrations above 40 ng/mL with a range of 40–80 ng/mL in the population is optimal for disease prevention and reducing morbidities and mortality without adverse effects. The recommendation for individuals is to maintain serum 25(OH)D concentrations above 50 ng/mL (125 nmol/L) for optimal clinical outcomes. Insights from metabolomics, transcriptomics, and epigenetics offer promise for better clinical outcomes from vitamin D sufficiency. Given its broader positive impact on human health with minimal cost and little adverse effects, proactively integrating vitamin D assessment and supplementation into clinical practice promises significant benefits, including reduced healthcare costs. This review synthesized recent novel findings related to the physiology of vitamin D that have significant implications for disease prevention.

**Definitions of vitamin D that are used in this review:**

***(a).***  ***Hypovitaminosis D = Insufficient 25(OH)D levels***

Hypovitaminosis D, also known as vitamin D *insufficiency* (an ambiguous term), occurs when there are inadequate (sub-optimal) levels of vitamin D in the circulation to support its intended physiological and biological functions—defined as serum 25-hydroxyvitamin D (25(OH)D) concentration of less than 40 ng/mL. Because hypovitaminosis D exacerbates common disorders and increases vulnerability to other disorders, such as infections, it cannot be considered within the physiological range.

***(b).***  ***Severe vitamin D deficiency***

Vitamin D deficiency is defined as having significantly low 25(OH)D in the circulation—a concentration of less than 12 ng/mL. Persons will present with signs and symptoms of vitamin D deficiency, mainly from the neuromuscular and skeletal systems. It significantly worsens most diseases—and increases complications and deaths from cardiovascular disorders, cancer, infections, and septicemia.

***(c).***  ***Vitamin D sufficiency:***

Achieving vitamin D sufficiency is crucial for overall health and robust bodily functions. A serum 25(OH)D concentration above 50 ng/mL is necessary to overcome prevalent common disorders and diseases—the physiological range is between 40 and 80 ng/mL. From the public health point of view, the minimum serum 25(OH)D concentration for the population (or community vitamin D sufficiency) is recognized as 40 ng/mL.

## 1. Introduction

Most aspects of human health and well-being and its multifaceted role in disease prevention and overcoming infections depend on vitamin D’s biological and physiological functions [1]. Vitamin D is a unique nutrient that is supposed to be obtained through sunlight exposure, dietary sources, and supplements. It has become an essential component of public health. Both the synthesized and ingested vitamin D undergo metabolic processes in the body, ultimately generating its most active form, calcitriol (1,25(OH)_2_D) [2]. It regulates the body’s calcium and phosphorus requirements.

In addition to its well-understood functions in bone health, emerging research suggests that vitamin D has established broader implications for overall health and disease prevention. Vitamin D/calcitriol receptors (VDR = CTR) are present in most tissues and cells in the body, indicating its numerous physiological processes outside bone metabolism. The knowledge related to an area that has significantly advanced over the past decade is the immune system [1]. Vitamin D metabolites profoundly modulate immune function to facilitate defense against infections [3].

Vitamin D deficiency increases the susceptibility to certain infections, especially tuberculosis [4] and respiratory tract infections [5]. Maintaining sufficient levels of 25(OH)D leads to reduced risks of infectious diseases and downregulating inflammatory responses [6,7]. Vitamin D has also been implicated in preventing chronic diseases such as cardiovascular disease [8], diabetes [9], cancer [10], and autoimmune disorders [3,11,12].

Research has also shown a strong association between vitamin D deficiency and an increased risk of developing the conditions mentioned above. However, the mechanism varies between tissues/disease entities [13,14]. Overall, understanding the physiology of vitamin D and its implications for disease prevention encompasses its diverse roles in bone health, immune function, and various physiological processes throughout the body [6,15]. Ensuring adequate vitamin D status through sunlight exposure, dietary intake, and supplementation contributes to overall health and well-being.

### 1.1. The Rationale for This Study

In recent years, many publications have focused on vitamin D, primarily its extra-skeletal benefits. Given the broadness of the topic, the author searched several research databases using keywords related to vitamin D, physiology, biology, mechanisms, disease prevention, and vulnerability. Combining keywords narrowed the number of relevant manuscripts to a manageable quantity. The search encompassed databases such as PubMed, Medline, Web of Science, and EMBASE, focusing on clinical studies, randomized controlled clinical trials (RCTs), prospective clinical studies, and original and review articles. The author followed a similar methodology of systematic and narrative reviews [16], meticulously selecting pertinent references and justifying their inclusion based on their relevance to the topic. These were incorporated into the manuscript after a thorough review. The review aims to present recent findings related to the extracellular functions of vitamin D concerning the proactive use of vitamin D supplements for disease prevention.

### 1.2. Importance of Vitamin D for Human Health

Vitamin D deficiency is a widespread problem that affects individuals of all ages and ethnic backgrounds, yet it remains largely overlooked by global health authorities [17]. The primary vitamin D source for humans is exposure to ultraviolet B (UVB) rays from sunlight [18,19]. However, a sizable portion of the population worldwide lacks adequate exposure, leading to sub-optimal concentrations of 25(OH)D in the circulation (i.e., less than 40 ng/mL). Studies indicate that more than half of the global population has insufficient vitamin D levels, surpassing the prevalence of iron deficiency as the most common micronutrient deficiency [19,20]. This deficiency is particularly prevalent among individuals residing far from the equator, where sunlight is limited, and those living within 500 km of the equator due to sun avoidance behaviors [21,22,23,24].

The consequences of vitamin D deficiency can be profound, impacting various aspects of health and contributing to the development of numerous health conditions [20]. The lack of ultraviolet-B (UVB) rays (i.e., higher latitudes—living far from the equator) and the behavioral issue—avoiding sunlight (within 500 km of the equator due to harsh conditions) are the two most typical causes for global vitamin D deficiency. Addressing this issue requires increased awareness, education, and public health initiatives to promote safe sun exposure, dietary sources of vitamin D, and supplementation where necessary. 

### 1.3. Clinical Signs and Symptoms of Vitamin D Deficiency

Unlike other deficiency statuses, clinical signs and symptoms of vitamin D deficiency do not manifest until the levels fall below 12 ng/mL [6,25]. Such very low circulatory levels of vitamin D and 25(OH)D are below the effective threshold for active transportation of these precursors into renal tubular cells. However, this inefficiency is partly compensated by increased 1α-hydroxylase activity, attempting to produce a more hormonal form of calcitriol [2,26,27]. As seen in the case of rickets in children and osteomalacia in adults, when the generation of calcitriol lags, signs and symptoms of deficiency begin to manifest [28,29,30].

Symptoms include lethargy, difficulty rising from a seated position or the bed, limited ability to raise arms above shoulder level (i.e., shoulder-girdle myopathy), muscle and joint pain, increased falls [25,31], and a tendency to develop hypothermia [32]. Vitamin D deficiency contributes to generalized weakness and fatigue, impacting overall energy levels [25]. Common clinical signs and symptoms of vitamin D deficiency include muscle weakness (and accumulation of osteoid tissues in bone), impaired calcium absorption, bone pain [33,34,35], skeletal abnormalities such as deformities (rickets) in children, weakened bones (osteomalacia, scoliosis) in adults [25,36], and fractures that can be prevented with vitamin D [35]. Additionally, individuals may experience bone pain, especially in the back, hips, and legs [25], and are at an increased risk of fractures due to reduced bone density caused by impaired calcium absorption [35,37].

Studies have also reported a link between low vitamin D levels and symptomatic depression, impaired wound healing [38], thinning of hair and hair loss [39,40], atrophy of type II muscle fibers [41,42], hypocalcemic seizures in children [43], disruptions in the regulation of energy metabolism [44] and the immune system [12], and poor health [37]. It is worth noting that many of these symptoms are nonspecific and may overlap with other health conditions such as rheumatic disorders, fibromyalgia, and hypothyroidism [34]. Additionally, the extraskeletal body systems do not exhibit specific symptomatology; instead, they present with progressively increased risks or worsening of metabolic disorders (such as diabetes and obesity), cardiovascular and other systemic disorders, and heightened vulnerability to cancer and infections [45,46,47,48].

### 1.4. Current Recommendations and Vitamin D Status

Inadequate exposure to sunlight and sun avoidance behavior are the two predominant factors causing vitamin D deficiency worldwide. Inefficient conversion of 7-dehydrocholesterol into pre-vitamin D and then to vitamin D in the skin can be caused by (i) deficient intake of dietary vitamin D or not taking supplements, (ii) gastrointestinal issues leading to impairment of vitamin D absorption, (iii) increased catabolism of vitamin D following intakes of medications that enhance the activity of some cytochrome P450 enzymes, (iv) insufficient expression or activity of CTR (e.g., due to lack of cofactors) or genetic abnormalities of vitamin D receptors, (v) activation failure of vitamin D and its metabolites as in liver cell or renal tubular cell impairment, and (vi) rare genetic disorders.

Vitamin D deficiency has emerged as a global pandemic affecting individuals across all age groups and ethnic backgrounds, yet it remains largely overlooked by leading global health agencies. The human body relies on UVB rays from sunlight to synthesize vitamin D. However, insufficient exposure has resulted in more than half of the population having insufficient levels of 25(OH)D [49,50]. The consequences of vitamin D deficiency can have significant health implications, underscoring the importance of addressing this issue through increased awareness, education, and public health initiatives. Encouraging safe sun exposure practices, promoting dietary sources of vitamin D, and considering supplementation where necessary are crucial steps in combating this pervasive health concern [19].

Based on published data, the author estimated a global prevalence of vitamin D deficiency affecting approximately 4.9 billion people sometime during the year [51,52]. Unsurprisingly, older studies using an outdated definition of 20 ng/mL as vitamin D deficiency by the Institution of Medicine (IoM) [53] estimated the prevalence as over one billion [54,55]. The signs and symptoms associated with vitamin D deficiency can be alleviated by ensuring the appropriate dose of vitamin D is taken regularly, at the right frequency. For musculoskeletal diseases, improvements are observed when serum 25(OH)D concentrations reach around 20 ng/mL [56], which was the basis for the IoM report [53]. However, metabolic disorders require higher circulatory levels exceeding 40 ng/mL [57,58]. Examples include infections, autoimmunity [15,59], and cancers [60,61,62], which may require even levels above 50 ng/mL [6,15,63,64]. 

### 1.5. Vitamin D Dose Recommendations

Little vitamin D is present in natural food; thus, the dietary intake is minimal and cannot be relied upon for the majority [65,66]. In the absence of regular exposure to daily direct sunlight, casual exposure to the sun is inadequate for raising and sustaining the concentration of serum 25(OH)D [67,68]. Most governments and their appointed committees like the Scientific Advisory Committee (SCAN) in the UK, IoM, Food and Nutrition Board (FNNB), and USPTO in the USA, etc., continue to recommend doses of vitamin D of between 400 and 1000 IU/day, with 20 ng/mL as the minimum sufficient level. These low doses fail to raise serum 25(OH)D concentration by more than 6 ng/mL after vitamin supplementation [6]. This is grossly insufficient for those with vitamin D deficiency [69]. Therefore, such doses consistently fail to raise serum 25(OH)D concentrations above the minimum therapeutic level recommended above [69].

Raising serum 25(OH)D concentration above 20 ng/mL benefits the musculoskeletal system but not others [53,70]. Governments and their appointed committees’ recommended doses of vitamin D are primarily (of less than 1000 IU/day) aimed to prevent rickets in children and osteomalacia in adults [14,67,71]. However, this does not help any other body systems or disease conditions. Therefore, it is paramount to use adequate doses of vitamin D (preferably body-weight-based doses) [49,72] to increase serum 25(OH)D to the desired concentrations. 

For busy healthcare workers, it is difficult to remember the different doses of vitamin D and serum 25(OH)D concentrations needed for various diseases. Therefore, irrespective of age and body weight, when laboratory measurements are affordable and available, it is rational to maintain serum 25(OH)D concentrations above 40 ng/mL [73,74], preferably above 50 ng/mL—a range between 50 and 80 ng/mL [56,69].

Notably, the administered vitamin D vs. dose–response and serum 25(OH)D concentration is not linear [75,76,77]. Clinical research published on extra-skeletal disorders over the past decade is more favorable for having a higher minimum serum 25(OH)D concentration than 30 ng/mL recommended by the American Endocrine Society [78]. The primary rationale is that maintaining higher circulatory serum 25(OH)D concentrations leads to better short-term responses and longer-term clinical outcomes for more diseases [58,75].

Most scientific societies’ recommendations typically advocate maintaining serum 25(OH)D concentrations above 30 ng/mL [15], which is still insufficient [49,56,73,79,80]. This contrasts with the government’s recommendation of 20 ng/mL as adequate [53,81]. In contrast, higher vitamin D intakes, and serum 25(OH)D concentrations are necessary to overcome conditions such as cancer [60,61,62], autoimmune diseases [12,63], and infections to achieve and sustain serum levels above 50 ng/mL (discussed below) [56]. This highlights the potential inadequacy of current recommendations, which may be outdated in light of recent research [15].

A broad search of databases using keywords of vitamin D, clinical studies, and RCTs illustrated that while most studies have reported significant benefits from vitamin D supplementation, approximately 15% of clinical publications have inconsistent or negative results—i.e., lack of beneficial effects. Detailed evaluations revealed that most negative findings often stem from design flaws [49,56]. This is vivid in those studies funded by pharmaceutical companies, where vitamin D was administered as a drug intervention or piggybacked on a pharmaceutical RCT. Notably, most clinical research studies have focused on using vitamin D as a treatment rather than for disease prevention. Vitamin D’s primary benefit is that it is a micronutrient, yet its prophylactic use to prevent diseases was neglected. Therefore, future research should emphasize the preventive aspect of vitamin D supplementation to harness its health-promoting effects fully.

## 2. Generation of Vitamin D

The portion of calcitriol synthesized and secreted to the blood from the kidneys acts as a hormone that regulates calcium and phosphorus metabolism. It plays a crucial role in muscular skeletal health. It enhances the absorption of calcium and phosphorus from the intestines, promoting deposition in bones and maintaining bone mineralization [82]. Meanwhile, gross vitamin D deficiency leads to conditions like rickets in children and osteomalacia in adults, characterized by weakened and brittle bones [35,37,68].

### 2.1. Synthesis of Vitamin D

**Figure 1:**
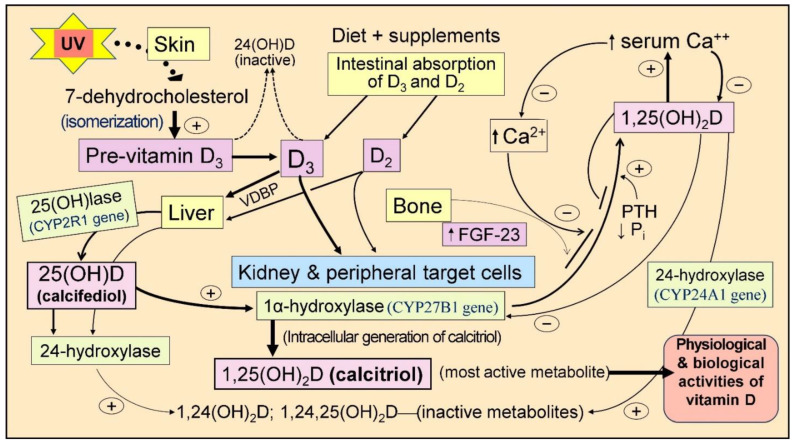
Illustrates the pathway for the generation of vitamin D_3_ from 7-dehydrocholesterol (7-DHC) following exposure to UVB rays. The activation of vitamin D to its metabolites, 25(OH)D (calcifediol) and 1,25(OH)_2_D (calcitriol), is highlighted, including its 24-hydroxy, inactive catabolic metabolites. Critical organs responsible for vitamin D generation/metabolism and the parathyroid hormone (PTH)-mediated regulation of serum ionized calcium (Ca^2+^) levels are illustrated. The typical activation route for skin-derived and oral/dietary vitamin D forming 1,25(OH)_2_D is depicted. While 25-hydroxylase activity occurs mainly in the liver, the conversion of vitamin D and 25(OH)D to 1,25(OH)_2_D via the 1α-hydroxylase enzyme occurs in renal tubules and peripheral target cells of vitamin D. The figure also demonstrates the control of serum Ca^2+^ levels through intestinal absorption, bone turnover, and PTH-mediated renal handling (+ upregulation and − downregulation) (Fibroblast growth factor-23 = FGF-23; UVB = Ultraviolet B rays; VDBP = Vitamin D binding protein).

While diet contributes some vitamin D_3_ (cholecalciferol) and D_2_ (ergocalciferol), the amount is typically insufficient [83]. Evolutionarily, most human vitamin D requirements are anticipated to be met through skin synthesis. However, as deliberate sun exposure has declined, the necessity for increased supplement intake has become unavoidable [2]. Skin exposure to ultraviolet B spectrum (290–315 nm) (UVB) causes a photolytic conversion of the 7-dehydrocholesterol (7-DHC) to pre-vitamin D_3_, which then undergoes a thermally induced isomerization to form pre-vitamin D [84] (Figure 1). 

Most pre-vitamin D_3_ is synthesized in the epidermis near the dermal capillary bed. Therefore, the skin surface temperature and its changes are unlikely to impact the formation rate of vitamin D_3_ in the skin [85]. The efficiency of this process depends on various factors, including skin melanin content, UVB exposure (duration, intensity, time of day, and season), UV-blocking products or clothing use, skin conditions such as scars, and age-related factors [86]. Figure 1 illustrates the sequence of vitamin D metabolite generation.

In individuals with fair skin, approximately 30–60 min of exposure, with about one-third of the upper body exposed to direct sunlight (while protecting the eyes and face from UV/sunlight), between 10:30 A.M. and 1:30 P.M., can yield up to 10,000 IU of vitamin D [87]. It is important to note that the skin has a built-in feedback mechanism preventing excessive vitamin D from sun exposure from entering the bloodstream, thereby avoiding hypervitaminosis D or hypercalcemia [88]. Besides synthesizing vitamin D, sunlight offers other advantages to human health [88]. However, minimal or no vitamin D synthesis occurs during early mornings, late afternoons, winter months, indoor sun exposure through double-glazed glass windows, or when clothing or sunscreen covers the skin [37,71,89,90].

### 2.2. Vitamin D Supplementation and Its Benefits

Except for sun-exposed mushrooms and fatty fish, food has little vitamin D. Therefore, the optimal way to obtain vitamin D_3_ is through daily safe exposure to ultraviolet sun rays. When direct sun exposure is not feasible, daily or weekly, supplementation can maintain physiological vitamin D concentrations in the circulation [49,56]. For communities with a high prevalence of vitamin D deficiency, targeted food fortification is a cost-effective way to alleviate it [71]. These approaches can ensure an adequate supply of vitamin D_3_ to maintain optimal vitamin D levels in the population—mean serum 25(OH)D concentrations above 40 ng/mL [84,91]. In individuals, maintaining levels above 50 ng/mL can bolster immunity, reduce illnesses and absenteeism, and enhance productivity. Moreover, a robust population immunity can help limit the spread of pathogenic microbial infections, including viral epidemics and pandemics such as SARS-CoV-2, consequently reducing hospitalizations and fatalities [92,93].

Large datasets and emerging evidence strongly support the diverse physiological functions of vitamin D mediated by calcitriol. These findings indicate that vitamin D should be utilized as a preventative and adjunct therapy in various common disorders, including sepsis and COVID-19 infection. Despite this, vitamin D is seldom included in clinical protocols or guidelines by leading health authorities or government recommendations to promote public health [20]. Furthermore, recommendations from medical and scientific societies often lack clarity and are contradictory and outdated [6,94].

However, public awareness regarding vitamin D and its beneficial effects on the immune system has increased since the COVID-19 pandemic [95,96,97,98]. This can be attributed partly to the persistent efforts of small groups of scientists despite negative publicity from pharmaceutical companies and health agencies. Notable examples include the clinical guidelines provided by the Front-Line COVID-19 Critical Care Alliance and informative articles on platforms like Substack and websites such as covid19criticalcare.com [99,100].

## 3. Physiology of Vitamin D

Over the past two decades, numerous non-classical actions of vitamin D beyond the musculoskeletal system have been documented [20,101,102]. However, peripheral target cells, like immune cells, rely on maintaining adequate vitamin D and 25(OH)D levels in circulation via diffusion of the two mentioned precursor molecules. For biological actions to occur, peripheral target cells must synthesize calcitriol intracellularly, as circulating calcitriol levels are relatively low (approximately 900-fold less than 25(OH)D) and thus do not enter these cells [6,56].

In addition, intracellular calcitriol magnesium and other cofactors are utilized during metabolic activities, immune cell activation, and signaling processes [6,103]. However, the government-recommended vitamin D of 400 to 800 IU/day is insufficient to maintain circulatory levels of D_3_ and 25(OH)D [56]. The entry of vitamin D_3_ and 25(OH)D from the circulation to peripheral target cells, like immune cells needing levels beyond 50 ng/mL [49,104,105], is necessary to maintain a robust immune system [56,63,106]. No evidence suggests that circulatory calcitriol enters target cells, such as immune cells, as the levels required are about two orders of magnitude lower for diffusion [49,63,107,108,109].

### 3.1. Non-Classical Actions of Calcitriol

Calcitriol engages in various regulatory and physiological homeostatic activities. Having physiological levels of two precursors of calcitriol ensures the efficient functioning of immune cells through genomic and autocrine intracrine mechanisms [104,105,106,110], thereby reducing the risks of cytokine storms and complications such as acute lung injury leading to ARDS from SARS-CoV-2 infection [111,112,113,114,115]. Individuals with severe vitamin D deficiency are particularly susceptible to such complications.

Vitamin D (a vitamin) and 25(OH)D (a metabolite of vitamin D) are not hormones [2,7]. Meanwhile, 25(OH)D hydroxylation produces calcitriol in renal tubular cells, which enter the circulation; it exhibits hormonal properties in the muscular-skeletal target tissues [15,103]. Both vitamin D and 25(OH)D diffuse from the circulation into other target cells and occur primarily through a concentration-dependent diffusion [108,109]. These target cells contain enzymes, 1α-hydroxylase and 25-hydroxylase, responsible for generating calcitriol and calcitriol (vitamin D) receptors (CTR/VDR) [6].

**Figure 2:**
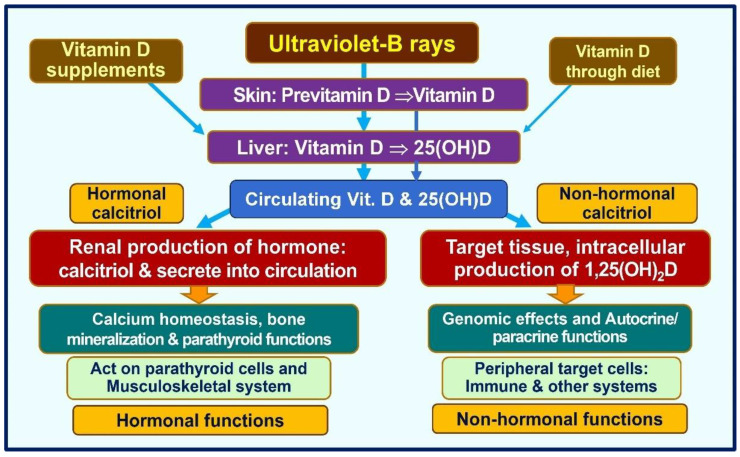
The essential pathways of acquiring vitamin D in humans are illustrated. The figure demonstrates key functional differences between the circulatory hormonal form of calcitriol (the hormonal form) that is generated in renal tubular cells vs. the intracellularly generated calcitriol in peripheral target cells, such as immune cells (modified from Wimalawansa, SJ., 2023 [7]).

Vitamin D signaling intracellularly (autocrine/intracrine) is crucial for multiple physiological activities, including stimulating and synthesizing intrinsic defensive compounds against microorganisms such as cathelicidin [116] and defensins [117], which have crucial anti-microbial activities [118]. In addition to directly binding to and destroying pathogens, cathelicidin also acts as a secondary messenger, enhancing vitamin D-mediated reduction in inflammation during infection [119]. Calcitriol also stabilizes tight junctions of epithelial cells of the respiratory tract and vascular system. This protects fluid leakage and viral dissemination into soft tissues [119,120]. Figure 2 illustrates fundamental differences between the hormonal and non-hormonal forms of calcitriol and the related generation of calcitriol.

### 3.2. Activation of Vitamin D/CTR

The steroid receptor CTR is present in nearly all cell types of the human body, especially in immune cells [121]. When calcitriol binds to the CTR in the cytosol, it forms a heterodimer with the retinoid-X receptor. This complex then translocates into the nucleus, binding to DNA [122]. This binding initiates the recruitment of coactivator or corepressor proteins and transcription factors, ultimately regulating gene expression. This process can influence the transcription of numerous genes, estimated to be over 1200 [123].

The intracellular generation of calcitriol in target cells (immune cells, colon cells, breast cells, etc.) is crucial for vitamin D-related physiological functions [124,125]. This process is essential for autocrine and paracrine functions of vitamin D and its DNA interactions—genomic actions [126,127]. These intracellularly generated calcitriol gets metabolized and does not enter the circulation. One exception is the calcitriol synthesized within overactivated macrophages in granulomatous tissues, such as sarcoidosis and granulomatous tuberculosis, which can spill over to circulation [128]. This overflow of calcitriol into the bloodstream could lead to hypercalcemia, although this is uncommon.

### 3.3. Genetic Influences on Vitamin D/CTR

Genetics plays a role in determining certain aspects of skeletal development and the potential peak bone mass. However, vitamin D, dietary calcium, physical activity, and hormonal status also significantly influence and modulate peak bone mass, bone density, and skeletal mineral content accrual [129,130]. Vitamin D deficiency triggers increased parathyroid hormone (PTH) secretion, leading to secondary hyperparathyroidism; this is for maintaining serum ionized calcium concentration [131].

Elevated PTH levels resulting from low dietary calcium or, more commonly, vitamin D deficiency contribute to heightened bone turnover and gradual loss of bone mineral content [132], increasing fracture risk [133]. In addition, insufficient circulatory calcitriol reduces intestinal calcium absorption, while urinary calcium excretion is increased due to secondary hyperparathyroidism [131,134].

### 3.4. Vitamin D, 25(OH)D, and 1,25(OH)_2_D

Serum 25(OH)D concentration reflects both cutaneous production of vitamin D from ultraviolet B exposure and dietary intake from food and supplements. While it stands as the best indicator of vitamin D status, it does not directly reflect the amount of vitamin D stored in the body, which can vary depending on factors like muscle and fat mass. In epidemiological studies and clinical practice, serum 25(OH)D levels have become the primary marker for assessing vitamin D status. With daily administration of D_3_, measurement of cholecalciferol would be better [135], but its availability is limited. However, there is a risk of misclassifying individuals’ vitamin D status due to differences in half-lives—approximately one day for vitamin D compared to two to three weeks for 25(OH)D [136]. Some researchers advocate measuring both serum vitamin D and 25(OH)D concentrations in epidemiological studies, although this approach significantly increases costs [135].

**Figure 3:**
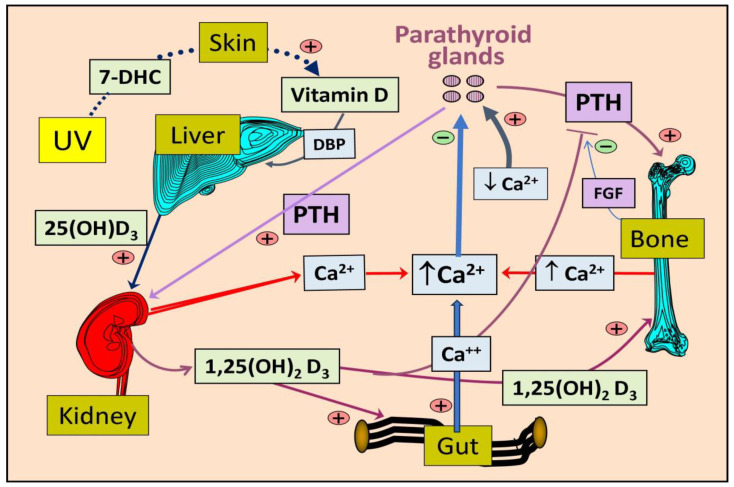
Illustrates the pathways for the synthesis of vitamin D and its activation into 25(OH)D in the liver and 1,25(OH)_2_D in the kidneys, as well as the role of parathyroid hormone (PTH) in the maintenance of ionized calcium (Ca^++^) in the circulation (7-DHC, 7-dehydrocholesterol).

In contrast, with a few hours of circulatory half-life, circulating serum 1,25(OH)_2_D concentrations do not reflect vitamin D status or the amount of vitamin D or 25(OH)D stored in the body and thus should not be used as a measure to assess vitamin D status. Serum calcitriol concentrations may be lower in individuals with renal failure, due to the progressive failure of renal cells to convert 25(OH)D to 1,25(OH)_2_D. Additionally, rare genetic disorders with abnormalities in the CYP2R1 gene, which is involved in vitamin D metabolism, can result in a relative deficiency of the 25-hydroxylase enzyme, leading to low calcitriol levels [137]. These conditions are typically associated with low 25(OH)D concentrations, hypocalcemia, and secondary hyperparathyroidism. Figure 3 illustrates the major organs involved in vitamin D metabolism in conjunction with PTH-mediated serum ionized calcium homeostasis.

### 3.5. Intracellular Synthesis of Vitamin D and Binding to CTR

Vitamin D is crucial in maintaining calcium and phosphate homeostasis [138] and skeletal mineralization, essential for overall human health [139,140]. There are two primary isoforms of vitamin D: vitamin D_2_ and vitamin D_3_. Vitamin D_3_, the primary source in humans, is synthesized in the skin upon exposure to ultraviolet B (UVB) rays. Through a series of enzymatic reactions [83], 7-dehydrocholesterol in the skin is converted to pre-vitamin D, which is then isomerized to form vitamin D [141,142].

Fat-soluble vitamin D binds to vitamin D-binding protein (VDBP) before entering the bloodstream via the skin capillary system. Once in the bloodstream, it undergoes 25-hydroxylation in the liver via 25-hydroxylase enzymes (from the CYP2R1 gene) [143] to form 25(OH)D [144], also known as calcifediol. Subsequently, 25(OH)D is further hydroxylated at the 1α-position by the 1α-hydroxylase enzyme (CYP27B1), predominantly within renal tubular cells [145,146], forming the hormonal form of vitamin D 1,25(OH)_2_D or calcitriol.

However, calcitriol is also synthesized in peripheral target cells, including immune, colon, breast, and prostate [147]. This local production is influenced by various signals from several sources, such as cell-surface Toll-like receptors [148,149]. Depending on the tissue and the signaling received, the genomic interaction of 1,25(OH)_2_D with its receptor, the calcitriol/vitamin D receptor (CTR), modulates (enhances or suppresses) the transcription of over 1200 essential genes [7,150]. In mammals, hepatic P450 cytochrome enzymes catalyze the formation of calcifediol via 25-hydroxylation and calcitriol via 1α-hydroxylase in microsomes [151] (Figure 1). The CYP24A1 gene expresses a 24-hydroxylase enzyme responsible for inactivating vitamin D and its metabolites [152].

Inclusion of other micronutrients, such as magnesium, zinc, and selenium, vitamins K_2_, A, and C, resveratrol, and, combined with essential fatty acids, such as omega-3, would enable the maintenance of a robust immune system [153,154,155,156] and health; these are crucial to overcoming infections [56]. Maintenance of the circulatory 25(OH)D concentrations above 50 ng/mL facilitates the sufficient generation of calcitriol intracellularly in immune cells (and other peripheral target cells) that enhance the expression of a series of anti-microbial peptides and neutralizing antibodies and signaling molecules [56]. These steps require proper concentrations of intracellular calcitriol significantly higher than in circulation. Calcitriol synthesis requires the availability of its precursors, vitamin D and/or 25(OH)D, within target cells [49]. This initiates autocrine/intracrine and paracrine signaling, in addition to multiple genomic mechanisms, enhancing the expression of anti-inflammatory cytokines from lymphocytes and macrophages and suppressing inflammatory cytokines, enabling the subduing of inflammation and oxidative stress, and preventing cytokine storms [97,157].

### 3.6. Binding of Calcitriol to Its Receptors (CTR)

The binding of calcitriol to the CTR triggers the activation of heterodimerization between the receptor and the retinoid X receptor (RXR) [145,158]. This heterodimer complex then translocates into the nucleus, binds to vitamin D response elements, and initiates modulation and transcription of genes. In addition to this classical genomic pathway, calcitriol modulates second messenger systems. It affects the host’s and neighboring cells’ biological functions through non-genomic pathways, such as membrane effects and autocrine/intracrine and paracrine signaling. Furthermore, calcitriol influences growth factors, cytokines, and the renin–angiotensin axis [159,160].

The functional calcitriol–CTR–RXR complex binds to the vitamin D response element in the promoter region of its target genes [145,158]. Subsequent downstream actions recruit transcription factors, coactivators, or corepressors, thereby regulating mRNA expression from target genes and modulating their functions. These functions include calcium and phosphate metabolism, neurotransmission, immunoregulation [1], and hormone secretion in target endocrine cells. For instance, this pathway is responsible for the slower genomic effects of increasing phase 2 insulin secretion in glucose-stimulated insulin secretory responses [161], thereby linking hypovitaminosis D to relative insulin deficiency. This example underscores the intricate mechanisms associated with the actions of vitamin D.

### 3.7. Vitamin D Is a Crucial Regulator of Calcium Homeostasis

Serum 25(OH)D measurement is crucial for determining an individual’s vitamin D status. Circulating 25(OH)D serves as the substrate to produce the hormonal form of calcitriol in renal tubules and calcitriol in extra-renal target tissues, which is essential for genomic and autocrine/paracrine signaling [49,125]. However, the precise quantities involved remain unknown [162]. There is no evidence to suggest that this component of calcitriol synthesizes in peripheral tissue cells and enters circulation [56], except in cases of overactive macrophages in granulomatous cells [6,163].

Therefore, the hormonal actions of calcitriol are exclusively dependent on renal tubular cell synthesis. Consequently, impaired renal function can significantly negatively affect the musculoskeletal system and calcium homeostasis due to the deterioration of renal tubular cell functions. Therefore, individuals with impaired renal functions must be supplemented with calcitriol or 1α-vitamin D analogs. The regulation of 1α-hydroxylase in renal cells is modulated by parathyroid hormone (PTH) but not in peripheral target cells [6]. In contrast, 25(OH)D and 1,25(OH)_2_D are inactivated through the 24-hydroxylase enzyme expressed from the CYP24A1 gene.

The coordinated actions of 1,25(OH)_2_D in conjunction with PTH and fibroblast growth factor-23 (FGF-23) tightly regulate serum ionized calcium (Ca^2+^) concentrations and phosphate (Pi) homeostasis [143,164,165]. This tight control of serum ionized calcium is crucial for numerous physiological functions, including enzymatic activities, hormone synthesis and release from endocrine glands (e.g., PTH and insulin), intestinal calcium absorption, DNA repair, mitochondrial energy generation, and the promotion of skeletal mineralization and microarchitectural integrity [165,166]. Fluctuations in ionized calcium in the blood can have detrimental effects on survival.

### 3.8. Maintenace of Calcium Homeostasis

Blood calcium homeostasis is maintained through various mechanisms, including promoting intestinal calcium absorption, calcium mobilization from bones (mediated by parathyroid hormone and osteoclasts), and calcium conservation by the kidneys. These biological actions, which involve osteoclasts and osteoblasts, help regulate mineralization and bone turnover to stabilize serum-ionized calcium and phosphorus concentrations [83,146,167]. Additionally, calcitriol and serum ionized calcium levels rapidly adjust intestinal fractional calcium absorption in local cells. This adjustment also affects concentrations of calcium-binding proteins like calbindin, reflecting the fine-tuning actions of 1,25(OH)_2_D [88,168,169].

Circulating PTH and hormonal calcitriol, directly and indirectly, influence the expression of genes like CYP2R1, which encodes 25-hydroxylase, and CYP24A1, which encodes 24-hydroxylase enzyme, to regulate serum ionized calcium levels [6]. Conversely, ionized calcium levels also impact the synthesis and release of PTH and the activity of 24-hydroxylase [170,171]. Consequently, vitamin D deficiency disrupts calcium homeostasis and phosphate metabolism at various stages. Moreover, chronic hypovitaminosis D can delay skeletal maturation and calcification, accumulating unmineralized bone tissue known as osteoid [170,172,173,174,175,176]. Clinically, this presents as rickets in children and osteomalacia in adults [146,167].

## 4. Key Physiological Functions of Vitamin D

Vitamin D is crucial in facilitating the intestinal absorption of calcium and phosphorus, promoting skeletal mineralization, enhancing resistance against bacteria and viruses, modulating inflammation and the immune system, and controlling cell growth. Over the past two decades, there has been exponential growth in our understanding of the additional biological and physiological functions of the vitamin D axis.

### 4.1. Tissue-Specific Regulation of CYP27B1

The molecular structures of cloned CYP27B1 are identical in renal and extra-renal tissues [177,178]. Functionally, the activity of CYP27B1 in target cells regulates processes such as cell proliferation, differentiation, immune functions, and hormone secretion, but it is not directly involved in bone mineral metabolism. The latter depends on the endocrine effects of 1,25(OH)_2_D. Consequently, the serum concentrations of 25(OH)D required for bone mineral metabolism are lower. This suggests the essential physiological roles of extra-renal tissue activation of calcitriol. However, the regulation of CYP27B1 expression and activity in extra-renal target tissues differs from that of CYP27B1 in the kidney.

One of the most abundant drug-metabolizing P450 cytochrome enzymes, CYP3A4, is present in the liver. It is constitutively expressed in hepatocytes and small intestines [179] and is crucial in metabolizing various toxins and pharmaceutical agents [6,180]. Interestingly, 1,25(OH)_2_D plays a feedback role in enhancing the transcription of CYP3A4 through a mechanism mediated by the calcitriol receptor (CTR) [181,182]. 

Serum 25(OH)D concentration necessary to reduce disease risks varies depending on the tissue or the specific disease [20]. For instance, to resolve conditions like rickets and osteomalacia, serum 25(OH)D concentrations between 15 and 20 ng/mL are typically sufficient, as the CTR in skeletal tissues is highly sensitive to activated vitamin D metabolites [182]. However, for the prevention of cardiovascular diseases, autoimmune disorders [12,63], and cancer [60,61,62], and the reduction of all-cause mortality [183,184], higher concentrations of serum 25(OH)D are generally required [20,183,185].

### 4.2. Tissue-Specific Thresholds of Vitamin D

**Figure 4:**
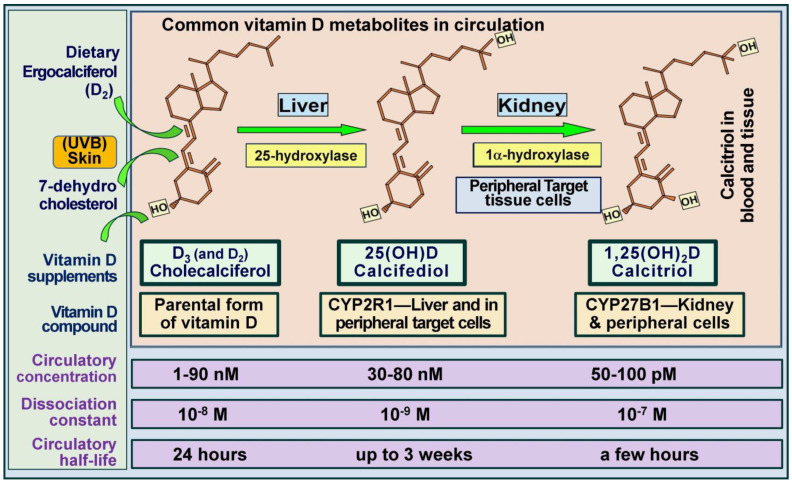
The figure depicts the structures of the three most common vitamin D metabolites, highlighting sites of generation, specific hydroxylating enzymes, and their average concentrations in the bloodstream. With daily supplementation or sun exposure, D_3_ and 25(OH)D concentrations remain similar and in equilibrium. Notably, while circulatory concentrations of D_3_ and 25(OH)D_3_ are in the nanomolar range, 1,25(OH)_2_D (calcitriol) is present in picomolar amounts—approximately 900-fold lower (modified from Bickel, D [186] and Wimalawansa, 2022 [49]).

Until a few years ago, the clinical importance of circulating vitamin D, 25(OH)D, and 1,25(OH)_2_D concentrations was underestimated [51]. While calcitriol has the most potent biological functions, all three metabolites participate in different physiological activities [6]. For example, vitamin D directly stabilizes epithelial and endothelium [56]. Vitamin D and 25(OH)D enter renal cells and all peripheral target cells to form calcitriol [6]. Figure 4 illustrates the differences between vitamin D and its two metabolites.

Due to the unfamiliarity with the biology of vitamin D, some study investigators neglected to measure baseline 25(OH)D concentrations before enrolling participants into clinical trials. Consequently, vitamin D-sufficient individuals may inadvertently be included in study groups [15], representing a significant and recurrent design flaw in randomized controlled trials (RCTs) [187]. Furthermore, aside from diagnosing hypovitaminosis D, low serum 25(OH)D could be a marker for poor health and nutritional status [188].

Many studies have reported that elevated circulating 1,25(OH)_2_D levels or a lower ratio of 25(OH)D to 1,25(OH)_2_D serve as predictors of poor health outcomes in individuals with cardiovascular diseases (CVD) [189,190], particularly regarding vascular calcification [191] and heart failure, leading to premature death [13,192,193]. Additionally, circulating 25(OH)D concentrations show an inverse association with the severity of various disorders, including metabolic syndrome [194,195], immunity [196,197], autoimmune disorders [12,198], the ability to combat infections [95,96,98,199], and the progression of calcified plaque in coronary arteries [200].

### 4.3. Other Beneficial Effects of Vitamin D

Vitamin D status during pregnancy is crucial in clinical outcomes, including the risk of pre-eclampsia [201]. For instance, dysfunctions and abnormalities in vitamin D metabolism observed in trophoblasts and placental endothelial cells (involving enzymes CYP2R1, CYP27B1, CYP24A1, and proteins VDBP and CTR have been proposed as significant pathological factors contributing to pre-eclampsia [201]. Therefore, distinct tissue-specific expressions and metabolic irregularities of vitamin D are associated with certain human disorders and conditions.

In addition, various endocrine glands and cells, including pancreatic islets and parathyroid cells, also express CYP2B1 [202,203]. The autocrine/intracrine and paracrine effects (signaling) of 1,25(OH)_2_D in peripheral target cells present a significant therapeutic opportunity for developing new drugs to modulate physiological processes related to vitamin D. However, the success of such therapies hinges on a comprehensive understanding of the clinically relevant 25(OH)D concentrations needed in different target tissues for optimal health [204]. Achieving stable serum concentrations of 25(OH)D is essential for long-term optimal physiological functions.

Another potential explanation for tissue- and organ-specific effects, as well as ethnic differences in responses, could be the availability of “free” (unbound) vitamin D. A study reported similar concentrations of bioavailable, free 25(OH)D in whites and blacks despite significantly lower concentrations of total 25(OH)D and VDBP in the African Americans [205]. The authors propose that differences in the prevalence of common genetic polymorphisms between racial groups may account for this disparity [206].

**Figure 5:**
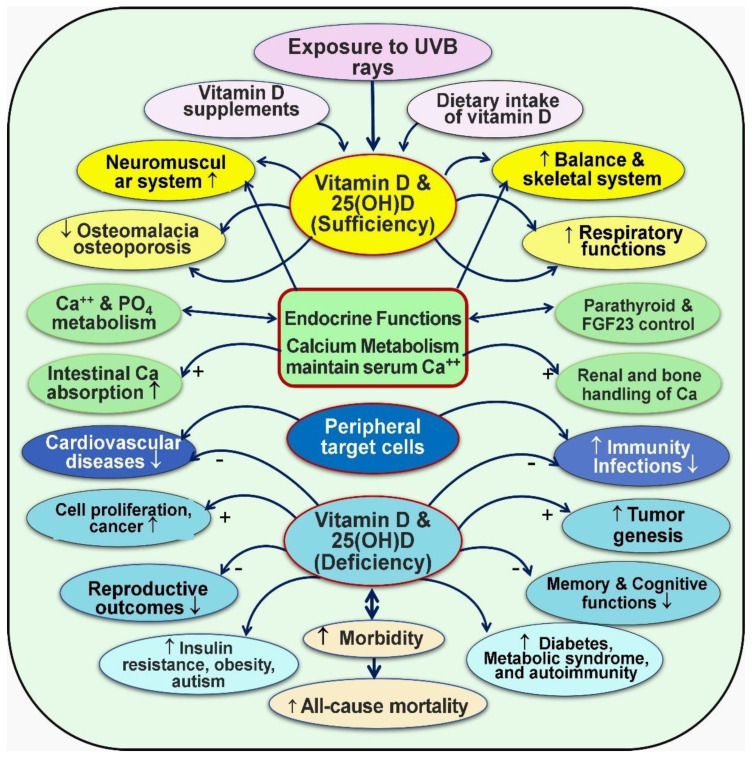
Broader functions of vitamin D (1,25(OH)_2_D): The figure illustrates both endocrine and non-endocrine functions that affect various cells and tissues in the body (modified from Wimalawansa, SJ, 2023; [15]) ↑ = Increased activity; ↓ = Decreased activity; − = Negative (reduced); + = positive (enhanced).

Concentrations of free 25(OH)D in the circulation and tissue fluids are regulated by vitamin D binding protein levels. In contrast, in target tissues and cells, such concentrations are regulated by local autocrine needs and intracellular 1α-hydroxylase activity. Figure 5 summarizes the essential physiological functions of hormonal calcitriol synthesized in renal tubular cells and non-hormonal calcitriol in peripheral target cells.

The PTH and the FGF23 modulate the renal production of calcitriol–Klotho endocrine system via the kidneys. Extra-renal synthesis of calcitriol is determined by the concentration of substrate, 25(OH)D, 1α-hydroxylase activity in target tissue cells, and by the catabolic enzyme 24-hydroxylase. Functional anomalies of vitamin D occur through various mechanisms, including CTR gene polymorphism and abnormalities of its CYP family of conversion enzymes.

### 4.4. Clinical Consequences of CTR, CYP27B1, and CYP2R1 Mutations

While 25-hydroxylase deficiency can cause rickets, such occurrences are rare [207,208,209]. Rickets can arise from mutations affecting the CTR gene encoding the 1α,25-dihydroxyvitamin D receptor, the gene encoding the vitamin D 1α-hydroxylase (CYP27B1; located on 12q13.1), and a microsomal vitamin D 25-hydroxylase (CYP2R1; located on 11p15.2) [210]. Mutations in CYP27B1 lead to 1α-hydroxylase deficiency, known as vitamin D-dependent rickets type 1 or hereditary pseudo-vitamin D-deficient rickets, while mutations in CYP2R1 result in 25-hydroxylase deficiency [137,211,212]. Despite the genetic differences, the phenotypic outcomes remain consistent.

CYP2R1 serves as the primary 25-hydroxylase enzyme in humans. Mutations in the CYP2R1 gene can result in genetically driven vitamin D deficiency, with inheritance patterns observed in some cases. Notably, mutations such as L99P and K242N in exon 2 of the CYP2R1 gene have been documented [209,213]. Such mutations can cause a significant reduction or complete loss of 25-hydroxylase activity [213], leading to a condition known as atypical vitamin D-deficient rickets (or vitamin D-dependent rickets type 1B) [213].

### 4.5. Health Economics of Vitamin D—Costs to Maintain Physiological Serum 25(OH)D Concentrations

Vitamin D is a readily available generic micronutrient that is accessible worldwide without the need for prescriptions. Among its various forms, D3 stands out as the preferred choice for supplementation, as it offers a cost-effective option. In the Western market, D_3_ supplements are typically priced at less than USD 8 per year’s supply. It can be utilized as an adjunct therapy in infections and metabolic disorders. For example, when used in SARS-CoV-2 infection, the cost is about USD 2 per person, compared to over USD 700 with anti-viral agents (https://c19early.org; accessed on 28 March 2024) [100,209,214]. Maintaining optimal circulatory levels of 25(OH)D, above 50 ng/mL, can be achieved through regular vitamin D supplements (e.g., 70–90 IU/kg body weight for a non-obese person [49] or through safe sun exposure. This could lead to sunlight potentially reducing the prevalence and severity of several common chronic diseases and certain acute illnesses like infections.

On average, addressing vitamin D deficiency incurs a cost of less than 0.1% of the expenses associated with investigating and treating exacerbating comorbidities and complications linked to vitamin D deficiency, such as those observed in COVID-19 [215]. For instance, the typical expenses for managing diseases associated with vitamin D deficiency, including diabetes, obesity, multiple sclerosis, and related complications, range from USD 5000 to USD 18,000 annually [216]. The cost–benefit ratio of prophylactic vitamin D averages approximately 1:10,000, indicating substantial potential benefits relative to costs.

The cost-effectiveness of addressing vitamin D deficiency enables individuals to enjoy longer, healthier lives with fewer illnesses and reduced expenses. Consequently, various entities and individuals with conflicts of interest, philanthropic organizations, prominent health organizations, and government-appointed committees persistently undermine the significance of vitamin D. This lack of acknowledgment is unsurprising, given their dependence on funding and benefits from pharmaceutical companies.

### 4.6. Repeated Supra-Pharmacologic Doses Are Unphysiological and Can Be Harmful

Large fluctuations in serum 25(OH)D concentration, particularly following high-dose therapy, could negatively affect bodily functions [125]. Supra-pharmacologic doses of oral vitamin D can lead to high peak serum 25(OH)D concentrations, accompanied by fluctuations in target tissue and intracellular concentrations. Similar fluctuations may occur when a large bolus of vitamin D is administered parenterally and cleared from the circulation within a few weeks [125], resulting in a peak followed by a rapid decline in serum concentration.

This phenomenon occurs partly due to the rapid absorption of vitamin D after administering large doses and the relatively shorter half-life of vitamin D compared to 25(OH)D in the circulation. As a result, there can be fluctuations in the bioavailable intracellular 25(OH)D concentration, as observed within prostatic cells [51,87]. This fluctuation could be one reason for the reported U-shaped curve observed with vitamin D therapy in prostate cancer patients.

Infrequent administration of high doses of vitamin D (e.g., 300,000 to 600,000 IU) administered at 6- to 12-month intervals [217] is not physiological and may lead to adverse clinical outcomes. Large doses of vitamin D administered biannually or annually [218] are unphysiological and thus not recommended [219]. One explanation is that higher transient serum 25(OH)D concentrations obtained with intermittent bolus high doses induce the 24-hydroxylase enzyme [220], causing a rapid decline of active forms in the circulation, 25(OH)D and 1,25(OH)_2_D concentrations over time [221].

Supra-pharmacologic doses of vitamin D administered at intervals of less than a month [222,223,224] could lead to negative clinical outcomes, such as increased falls and resulting fractures, as demonstrated in some improperly designed clinical trials [217,225]. Therefore, vitamin D supplements should be given at less-than-monthly intervals and preferably administered daily, especially in RCTs [226].

### 4.7. Common Adverse Effects Following an Overdose of Vitamin D

Adverse effects following vitamin D overdose are infrequent. Toxicity may occur when serum 25(OH)D concentration exceeds 150 ng/mL [49,56,227]. Most reported cases were due to mistaken or inappropriate doses taken for prolonged periods [228,229,230,231,232,233,234]. Signs and symptoms of vitamin D toxicity are primarily related to elevated blood-ionized calcium levels and hypercalciuria. The former include loss of appetite and weight loss, nausea and vomiting, constipation, weakness, fatigue, disorientation and mental cloudiness, cardiac arrhythmia, and possibly increased mortality [231,235].

Hypercalcemia-associated hypercalciuria leads to excessive urination, thirst, dehydration, and the formation of renal stones. Vitamin D can interact with drugs at high doses, particularly those that modulate cytochrome P450-3A4 (CYP3A4) [230,232]. Additionally, vitamin D can interfere with anti-convulsant and statin drugs, thiazide diuretics, verapamil, and digitalis agents [6].

### 4.8. Sustained Serum 25(OH)D Concentrations Are Necessary for Optimum Outcomes

Preventing vitamin D deficiency-associated diseases requires achieving and sustaining adequate serum 25(OH)D concentrations [236,237]. Moreover, obtaining clinical benefits necessitates different serum 25(OH)D concentrations for different diseases [20,90,238], including cancer [239] and type 2 diabetes mellitus [240,241], as well as reducing the risk of all-cause mortality [238,242,243]. It is necessary to sustain circulatory 25(OH)D concentrations above 40 ng/mL to mitigate the risks and severity of certain extra-skeletal disorders [20,73,244,245,246,247].

**Figure 6:**
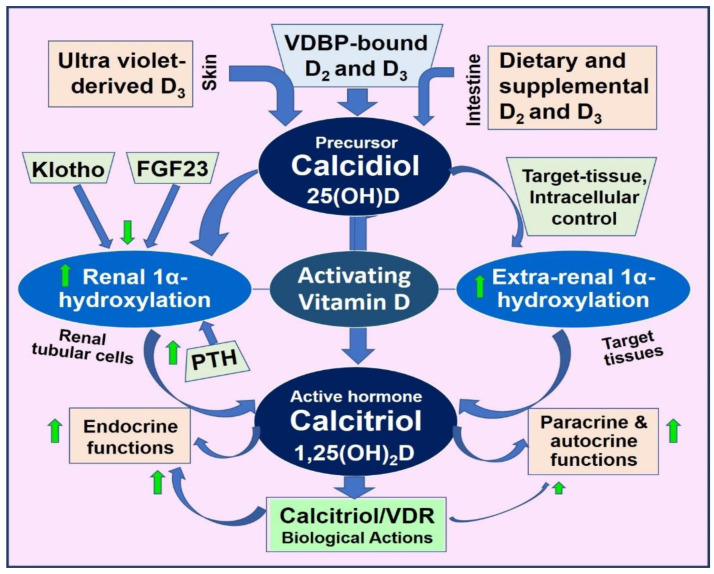
The illustration highlights the key activation steps and intricate yet vital interactions of vitamin D and its active metabolites, as well as their inherent feedback control systems. These mechanisms maintain circulatory ionized calcium concentrations, representing vitamin D’s fundamental endocrine function (Up arrow = up-regulated (increased activity); Down arrow = down-regulated (reduced functions)).

Much of the data supporting these findings are derived from ecological and observational studies. However, mounting evidence from recent RCTs report that elevated serum 25(OH)D concentrations are associated with increased health benefits [248]. Nevertheless, there is a lack of well-designed RCTs on vitamin D-deficient subjects, with vitamin D as a primary intervention to test specified diseases and vitamin D status, and that rely on serum 25(OH)D concentrations believed to be needed for the reduction of the risk of a specific disease. Endocrine functions and interactions with various diseases and disorders are illustrated in Figure 6.

### 4.9. Example Conditions Requiring Higher Serum 25(OH)D Concentrations

As with other physiological approaches, vitamin D supplements aim to achieve optimal serum 25(OH)D concentrations to maximize benefits while minimizing or avoiding adverse effects [20]. For most musculoskeletal disorders, including bone mineralization, a serum concentration above 20 ng/mL seems adequate [53,249]. In contrast, the current data support maintaining serum 25(OH)D concentrations between 40 and 80 ng/mL as optimal for most other disorders [20,83,90,238]. There are exceptions that certain conditions would improve by maintaining a higher serum 25(OH)D concentration [6].

Examples of these include infection [49,56,105], septicemia [250], and SARS-CoV-2 [93], which require maintenance of serum 25(OH)D concentrations above 50 ng/mL. In addition, sleep disturbances, chronic fatigue, post-COVID syndromes (with similar fundamentals), and chronic pain are managed better by maintaining serum 25(OH)D concentration above 50 ng/mL [106,251,252,253,254]. Metabolic disorders like diabetes [255,256], obesity [257], and osteoporosis, as well as autoimmune disorders such as multiple sclerosis [258], rheumatoid arthritis [259], and psoriasis [260], and certain types of cancers [243,248,261,262,263] as well as reduced all-cause mortality [183] require the maintenance of serum 25(OH)D concentrations above 60 ng/mL.

### 4.10. How Much Vitamin D Intake Is Necessary?

Even with a daily administration of 5000 IU/day of vitamin D_3_ in a vitamin D-deficient non-obese (~70 kg) adult, it could take from weeks to several months to raise serum 25(OH)D concentrations to therapeutic levels [264] and restoring robust immune functions. Doses below 3000 IU/day are unlikely to achieve the necessary therapeutic levels of serum 25(OH)D concentrations, even after one year, in those with vitamin D deficiency [6].

Even daily administration of a D_3_ dose of 5000 IU/day takes several months to increase serum 25(OH)D concentrations to the therapeutic levels in vitamin D-deficient persons to restore robust immune functions. Doses of less than 4000 IU/day in 70 kg adults with vitamin D deficiency would not raise serum 25(OH)D concentrations to therapeutic levels, even in the longer term. Likewise, although a bolus or upfront loading dose of 300,000 IU takes three to four days to raise serum 25(OH)D concentrations, it cannot maintain 25(OH)D levels for more than three months, and while the counter-regulatory effects, such as large boluses, are known to be effective for at least three months, such large bolus doses alone are unlikely to be effective in treating acute situations such as severe COVID-19 illness.

In addition to its role in adaptive immunity, hypovitaminosis D weakens the generation of neutralizing antibodies, impairs cytotoxic immune cell function, diminishes the effectiveness of memory cells and macrophages, and reduces immune responses following vaccination. Individuals with compromised immune systems often have severe chronic vitamin D deficiency and are particularly vulnerable to adverse effects from SARS-CoV-2 infection or immunization. This susceptibility may result in autoimmune reactions [12,63], systemic hyper-inflammation, and pathological oxidative stress, leading to severe complications and mortality. The high prevalence of hypovitaminosis D among older individuals contributed significantly to the pandemic’s impact in 2020, with COVID-19 disproportionately affecting those with severe vitamin D deficiency [52,216].

The recommendation of sub-optimal (standard-outdated) vitamin D doses for everyone, irrespective of their body weight (including obese individuals) and other factors affecting their serum 25(OH)D concentrations or the lack of serum 25(OH)D-based calculation of appropriate doses for individuals [49,56], has resulted in the administration of pediatric doses of vitamin D to adults, without benefiting the recipients. This manuscript also illustrates examples and circumstances where the efficacy and clinical necessity of rapidly increasing serum 25(OH)D levels (such as in emergencies like sepsis, SARS-CoV-2 infection, in ICU settings, etc.) and target serum 25(OH)D concentration required to address underlying illnesses such as infection or cancer are demonstrated.

### 4.11. Doses of Vitamin D Needed to Boost Serum 25(OH)D Concentration

The required vitamin D doses vary depending on the individual’s vitamin D status and whether the condition is acute or chronic. The latter is based on serum 25(O)H)D concentrations and/or the body weight (or body mass index–fat mass) [56,72]. The specific calculations have been published [49].

#### 4.11.1. Vitamin D Requirements for Chronic Conditions

In a person with vitamin D deficiency, the body-weight-based doses mentioned in Section 4.5 could take from weeks to months to raise serum 25(OH)D concentrations to the desired therapeutic levels [6]. In these situations, as described below, it is helpful to give an upfront loading dose (a bolus dose) to increase serum 25(OH)D concentrations within a few days instead of months [49,265].

Chronic long-term maintenance of circulatory 25(OH)D concentrations is crucial for the intracellular generation of calcitriol D_3_ [108,266,267]. If the serum 25(OH)D concentration is too low or the person has conditions requiring higher daily doses (unless presented with taking higher doses), they would benefit from one-time administration of high doses of oral vitamin D (a stat dose or split over a few days using 50,000 IU capsules, taken after a meal to facilitate absorption) to replenish vitamin D stores in the body [6,268,269]. When using high doses of D_3_, it is important to prevent triggering upregulation of 24-hydroxylase and downregulating intracellular signaling [6,108,270,271].

Fixed higher doses of vitamin D replacement, in the form of 50,000 IU D_3_ capsules, are widely available and economical for clinical use; they are also standardized and have satisfactory gastrointestinal absorption when taken after eating. The 50,000 IU capsules (60,000 IU in India) are consumed as a single dose or in divided doses to achieve doses ranging from 100,000 to 400,000 IU [6]. For most people (with an average weight of 70 kg), administering vitamin D requires a dose of 200,000 IU (increase or decrease based on body weight). This approach suits non-urgent, outpatient, and community setups to boost serum 25(OH)D concentrations in those with vitamin D deficiency. They are also useful in emergencies when serum 25(OH)D concentrations are not known [49,56]. Evidence supports the efficacy of high-dose vitamin D supplementation in raising serum 25(OH)D levels within a few days [6,64,272,273,274,275,276,277].

While a bolus or upfront loading dose like 300,000 IU can raise serum 25(OH)D concentrations within three to four days, it will not sustain therapeutic levels beyond three months. Furthermore, the counter-regulatory effects triggered by such large bolus doses could reduce the level faster, thus, with a shorter period of effective levels, if repeated higher doses are administered [278,279,280], making them even less effective in treating acute conditions such as severe COVID-19 illness [6]. Thus, it is important to continue daily or weekly doses of vitamin D after a bolus dose.

#### 4.11.2. Rapidly Increasing Serum 25(OH)D to Boost the Immune System in a Day

In emergencies like COVID-19, sepsis/infection, and other acute illnesses, it is vital to immediately increase the circulating D_3_ and/or 25(OH)D concentrations [49,125] to diffuse into peripheral target tissues for intracellular generation of calcitriol [49,281,282].

From generation in the skin or ingestion (Figure 1), vitamin D_3_ and D_2_ undergo 25-hydroxylation in the liver to form 25(OH)D (calcifediol), typically taking about 3–4 days. In contrast, calcifediol is already 25-hydroxylated and bypasses the liver, becoming available in circulation within four hours of administration [283]. This rapid availability offers several benefits, including rapidly boosting the immune system and enhancing other protective bodily functions within a day. As a result, calcifediol proves particularly valuable in medical emergencies such as COVID-19, sepsis, and acute conditions like Kawasaki disease, multisystem inflammatory syndrome, acute respiratory distress syndrome (ARDS), burns, and vitamin D deficiency during pregnancy.

The recommended oral dose of calcifediol is 0.014 mg/kg body weight [49]. With a single dose of calcifediol, it is advisable to administer a loading dose of vitamin D_3_ concurrently with or within the first week of calcifediol administration, which would allow a longer duration of raised serum 25(OH)D [56]. As with other regimens, it is essential to prescribe an appropriate daily or weekly dose of vitamin D to maintain serum 25(OH)D concentrations within the therapeutic range.

## 5. Discussion

The benefits of vitamin D extend beyond its well-established roles in calcium and phosphate homeostasis and the prevention and treatment of conditions such as rickets, osteomalacia, and bone loss. Based on recent data, vitamin D deficiency is defined by serum 25(OH)D concentrations below 40 ng/mL, a threshold below which various disorders may develop or worsen. The optimal physiological range is between 40 and 80 ng/mL, effective against 99.7% of these disorders. Concentrations below 40 ng/mL are associated with the worsening of many extra-skeletal conditions, including higher risks of falls, fractures, metabolic disorders, cardiovascular diseases, cancers, and increased all-cause mortality [183]. This occurs even in healthy individuals, highlighting the crucial role of vitamin D in public health and disease prevention [183]. The optimal functioning of vitamin D in genomic, endocrine, paracrine, and autocrine systems is essential for numerous physiological processes that keep people healthy. 

Based on recent data, vitamin D deficiency is defined by serum 25(OH)D concentrations below 40 ng/mL, below which certain disorders are initiated and worsened. The physiological (effective) range is between 40 and 80 ng/mL, effective against 99.7% of the disorders [6]. Concentrations below are associated with several extra-skeletal disorders and conditions like higher risk of falls, fractures, various illnesses, and increased all-cause mortality, even in healthy individuals [183], illustrating its crucial role in disease prevention—Public Health. The optimal functioning of the vitamin D endocrine, paracrine, and autocrine systems is crucial for numerous physiological processes. Furthermore, the beneficial effects of vitamin D extend beyond its established roles in calcium and phosphate homeostasis, as well as the prevention and treatment of conditions like rickets, osteomalacia, and bone loss.

Vitamin D adequacy can be accurately assessed only by measuring serum 25(OH)D levels; concentrations of 1,25(OH)2D do not reliably indicate vitamin D status. Recent data from a variety of studies support a decreased incidence of non-skeletal disorders, such as hypertension [255], diabetes [256,284], multiple sclerosis [258], rheumatoid arthritis [259], osteoporosis [285,286], certain types of cancers [243,261,263], all-cause mortality [183,184], and infections when serum 25(OH)D levels are maintained above 50 ng/mL [243,255,256,261,263,284,287,288,289]. However, it is worth noting that despite the presence of physiologic concentrations of calcitriol, increased risks of illnesses and reduced longevity can still occur, suggesting the involvement of other factors in optimal health. However, it is essential to acknowledge that not all researchers agree on the reported multiple non-classical benefits of vitamin D [287,288,289].

Implementing a public health strategy to raise the mean population serum 25(OH)D concentration above 30 ng/mL would incur costs less than 0.01% of the expenses associated with investigating and managing diseases like diabetes. However, the mere existence of policies is insufficient; these policies must be accompanied by effective measures to ensure individuals reach the target vitamin D status. This includes recommendations for safe sun exposure, food fortification strategies, and vitamin D supplementation guidelines [14,19,290,291,292]. Adhering to practical public health guidelines makes eliminating vitamin D deficiency cost-effectively feasible.

The typical daily dose of vitamin D for a non-obese 70 kg adult ranges between 4000 and 7000 IU/day, or 50,000 IU once or twice a month, based on the target blood levels and the body weight. These dosages enable approximately 97% of individuals to maintain their serum 25(OH)D concentrations above 40 ng/mL [83,90]. Given the biology of vitamin D and the need to maintain a steady state of D_3_ or 25(OH)D in the circulation [125], daily administration of vitamin D_3_ is preferred to infrequent administration as a preventative measure. In cases where adequate sunlight exposure is lacking, most individuals may need a daily oral intake of vitamin D supplement ranging from 5000 to 7000 IU/day for the maintenance of circulatory 25(OH)D concentrations above 50 ng/mL (125 nmol/L) to have a meaningful positive impact on health. Sustaining stable serum 25(OH)D concentration over the long term is essential for reducing disease incidence and all-cause mortality.

## Data Availability

Not applicable.

## Abbreviations

1,25(OH)_2_D	1,25-dihydroxyvitamin D
25(OH)D	25-hydroxy vitamin D
7-DHC	7-dehydrocholesterol
CTR	Calcitriol receptor (synonymous with vitamin D receptor; VDR)
MR	Mendelian randomization
PTH	Parathyroid hormone
RAS	Renin–angiotensin system
RCT	Randomized controlled clinical trial
RDA	Recommended dietary allowance
RXR	Retinoid X receptor
UVB	Ultraviolet B
